# 25 years of providing evidence on road safety interventions at the city level

**DOI:** 10.3389/fpubh.2024.1463878

**Published:** 2025-02-07

**Authors:** Katherine Pérez, Elena Santamariña-Rubio, Maria José López, Lucia Artazcoz, Josep Ferrando, Carlos Pastor, Adnan A. Hyder, Carme Borrell

**Affiliations:** ^1^CIBER Epidemiología y Salud Pública (CIBERESP), Madrid, Spain; ^2^Agència de Salut Pública de Barcelona (ASPB), Barcelona, Spain; ^3^Institut de Recerca Sant Pau (IR SANT PAU), Barcelona, Spain; ^4^Universitat Pompeu Fabra, Barcelona, Spain; ^5^Guàrdia Urbana de Barcelona, Barcelona, Spain; ^6^Milken Institute School of Public Health, George Washington University, Washington, DC, United States

**Keywords:** road safety, road traffic injuries, policy evaluation, evaluation, injury surveillance, public health, information system, health impact assessment

## Abstract

Road traffic injuries are a significant public health concern, ranking among the leading causes of mortality and disability-adjusted life years lost globally, especially among the young population. Traditionally, road safety has been approached predominantly from a healthcare standpoint, with limited interventions from a comprehensive public health perspective. In Barcelona, the Agència de Salut Pública (Public Health Agency) has been monitoring road traffic injuries and evaluating road safety interventions since the late 1990s. This paper explores how Barcelona has addressed road safety over more than two decades through a public health lens, emphasizing the evaluation of intervention effectiveness, highlighting key success factors, and addressing the challenges encountered. First, we describe the road traffic surveillance system, providing insights into the context of mobility and road safety interventions in the city since the late 1990s. Since then, 10 interventions have been evaluated, encompassing legislation policies (helmet law, motorcycle driving license, and the penalty points system) and infrastructure measures (speed radars, advanced stop lines for motorcycles, safe routes to school, 30 km/h zone, and red-light cameras), as well as a cost–benefit study of speed radars. Next, the paper quantifies the overall impact of road safety interventions by estimating the difference between the observed number of road traffic injuries and the expected number if no interventions had been implemented from January 2008 to December 2023, stratified by gender, injury severity, and mode of transport. Since 2008, injuries were prevented in more than 34,800 individuals, including approximately 1,000 severe injuries. Mode-specific analysis revealed that more than 4,700 pedestrians, 12,300 car users, and 3,200 moped users benefited from injury prevention measures, while the number of injuries among motorcyclists was 5,200 higher than expected. This article discusses key success factors, the pivotal role of public health in road safety, and outlines future challenges, providing valuable insights for cities aiming to adopt a comprehensive public health approach to address road safety concerns.

## 1 Introduction

Road traffic injuries are a well-known public health concern with more than 1.19 million fatalities each year. They are the leading cause of fatalities among 5- to 29-year olds and are the 12th leading cause of death among all age groups, resulting in more than 50 million people being injured or disabled (1). Two-thirds of these fatalities occur in individuals of working age (18–59 years), leading to significant health, social, and economic consequences that reverberate throughout society. More than half of all road traffic deaths occur among vulnerable road users, including pedestrians, cyclists, and motorcyclists (1).

Multiple strategies have been developed since the late 1970s, mainly at the national level. However, less attention has been paid to implementing urban-specific road safety strategies (2). Nonetheless, most of the population lives in cities, both in low-middle and high-income countries. In cities, due to traffic congestion and lower speed limits, the number of fatalities is typically low, but there is a high number of traffic crashes involving many non-severe injuries, frequently affecting vulnerable users such as pedestrians, cyclists, two-wheeled motorists, and other personal mobility vehicles such as scooters (3).

From a health standpoint, road safety has usually been addressed from the perspective of health, treatment at emergency departments, or hospital admissions, especially at sub-national levels (4). There are fewer experiences of public health organizations involved in road safety at small area levels, probably because the actions primarily depend on other sectors such as police or mobility departments.

Barcelona is a Mediterranean city with 1.6 million inhabitants, covering 101.35 km^2^, and a density of 15,992 people per km^2^. The city attracts more than 12 million tourists annually. Every working day, there are more than 6.10 million commuting trips in the metropolitan area, of which 72.3% are internal to the city (4.42 million), and the remaining are connections. On weekdays, 74.6% of the trips starting or ending in Barcelona and carried out by city residents are made using sustainable modes of transportation: 46.4% on foot, by bicycle, or personal mobility devices, and 28.2% by public transport. With more than 270,000 two-wheeled motor vehicles registered, the modal share of private vehicles is 25.4%, with motorized two-wheeled vehicles accounting for 7%. Specifically, walking and metro journeys account for a high percentage, together representing 55.5% of the total flow.

In the early 1990s, the Agència de Salut Pública de Barcelona created a road traffic information system, working in close collaboration with the police and the mobility department. This system allowed monitoring of road traffic injuries and the evaluation of road safety interventions to inform policies and decision-makers. Documenting the experiences of road safety in cities for more than two decades is important to help inform the global dialog on road safety. This paper aims to discuss how road safety has been addressed for more than 20 years in Barcelona city by a public health organization, with special emphasis on evaluating the effectiveness of 10 road safety interventions, highlighting key success factors, and addressing the challenges encountered in the process.

## 2 Context

### 2.1 Barcelona road traffic injury surveillance system

The information system initiated in the early 1990s has been enriched by various sources. In addition to local police data, the system incorporates information from other sources such as hospital emergency departments, deaths reported by the Institute of Legal Medicine and Forensic Sciences of Catalonia, the Mortality Register of Barcelona, and regular health surveys conducted in the city (5–7). Since 1997, comparable data have been accessible for monitoring the magnitude and characteristics of individuals injured in traffic collisions. These include details on the type and severity of injuries, sourced from hospital emergency departments and forensic reports. The city’s local police instituted a crash register, encompassing information on the circumstances of collisions, geocoded locations, vehicles, drivers, and injured individuals. This comprehensive effort was formalized through a collaboration agreement signed between the Agència de Salut Pública de Barcelona, the local police, and the mobility department.

Other information sources include: (a) the annual mobility survey (EMEF), which provides information on trips and travel time in the metropolitan area, including Barcelona city; and (b) annual average daily traffic data, which provide traffic volume data from most streets and allow vehicle-km estimates.

These data sources have enabled road traffic injuries to be characterized by gender, age group, type of road user and crossing, and time of the crash. They also allow identification of the nature of injuries, and their severity based on diagnoses [using the Abbreviated Injury Scale (AIS) and Injury Severity Score (ISS)], monitoring of traffic injury trends and calculation of indicators using various exposures, such as the number of inhabitants, vehicles, trips, hours traveling, and vehicle-km. These data sources have also enabled assessment of social inequalities in injury mortality (8). Table 1 shows the main indicators used to monitor road safety annually in Barcelona.

**Table 1:** Indicators used for monitoring road safety by the city of Barcelona.

**Road traffic collisions and victims** N. of collisions with victims N. of vehicles involved in collisions with victims N. of victims* N. of people injured or fatalities Number of people with minor injuries Number of people seriously injured Number of people seriously injured or deceased N. of people killed in 24 h N. of people killed at 30 days N. people hospitalized in Barcelona due to traffic injuries **Indicators**Collisions, Vehicles, and Victims involved: per 10,000 inhabitants per 100,000 veh-km per 1,000 registered vehicles for 1,000,000 trips for 1,000,000 h-traveling per 100,000 passenger-km

* Disaggregated by gender when data allows it.

The geographical location of collisions, as recorded by police using geocodes, has been essential for understanding their distribution and characteristics, as well as for identifying risk areas based on mobility exposure. This approach has facilitated the selection of specific areas to evaluate road safety interventions. In addition, it has enabled probabilistic record linkage of hospital, forensic, and police data (9), providing a deeper understanding of the nature and severity of injuries according to the circumstances of the crash and type of road user.

### 2.2 Traffic injuries in Barcelona 2002–2023

**Figure 1:**
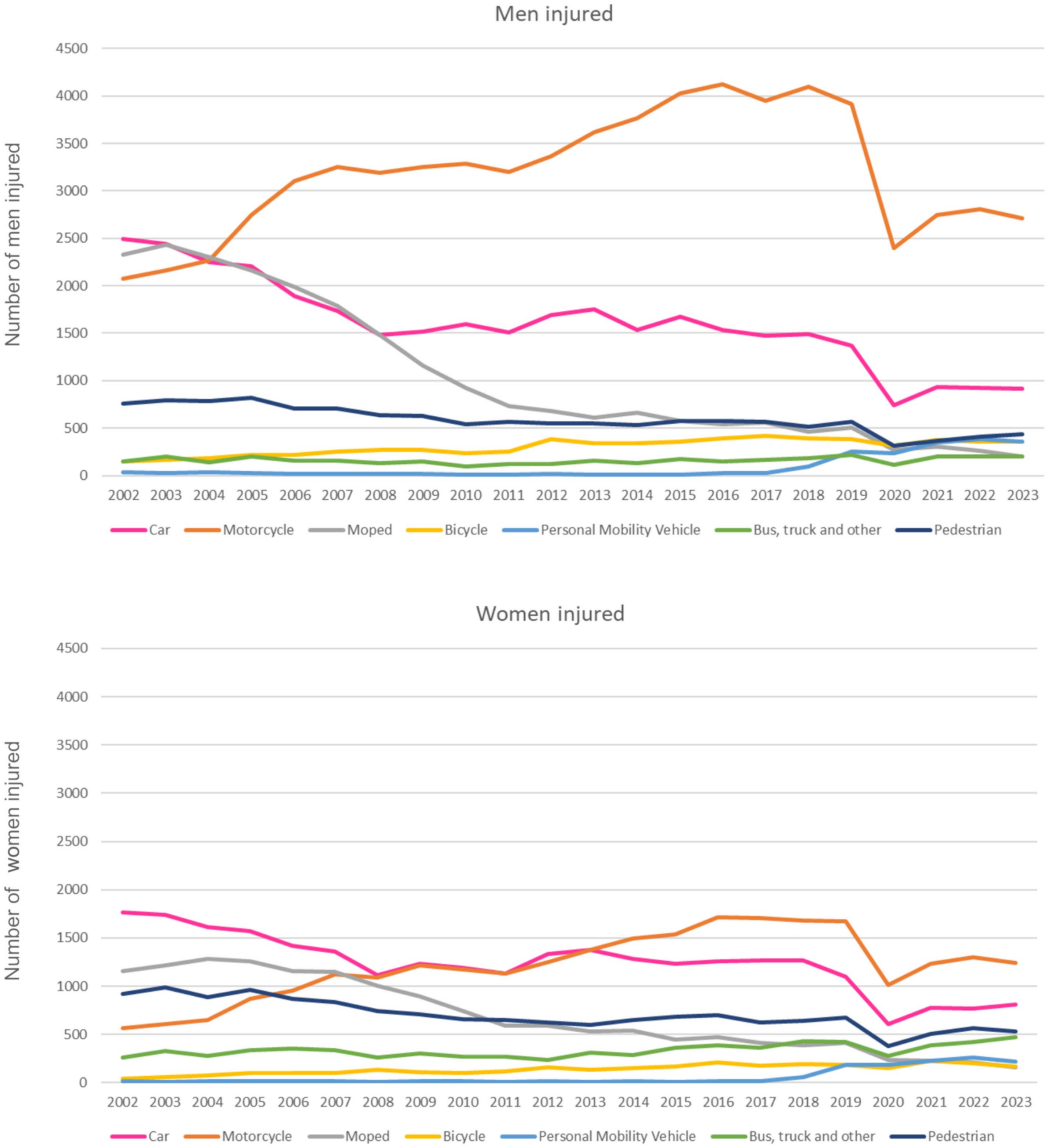
Road traffic injuries by mode of transport in Barcelona and gender. Barcelona 2002–2023.

Figure 1 shows the trend in road traffic injuries by mode of transport and gender from 2002 to 2023. According to the Accident Register of the Urban Guard of Barcelona, in 2023, there were 7,202 collisions resulting in 8,786 injured or deceased individuals, with 41% being women. Of these, 20 people died (two women and 18 men), and 225 were hospitalized due to serious injuries (78 women and 147 men). Almost half of the injured individuals were motorcyclists (34.5% women and 52.4% men), followed by car occupants (22.5% women, 17.7% men), and pedestrians (15.4% women, 8.4% men). Cyclists accounted for 4.7% of injured women and 6.9% of injured men. In addition, 12.3% of women and 2.8% of men were passengers on busses. Injuries involving personal mobility vehicles, such as scooters, accounted for 6.1% of injured women and 6.9% of injured men, with these figures showing a steady annual increase.

### 2.3 Barcelona road traffic policies

The Barcelona City Council launched its first Municipal Road Safety Plan in 2000–2003, demonstrating its commitment to designing policies to reduce traffic injuries and enhance mobility and road safety in the city. At the same time, the Barcelona City Council formalized a Mobility Pact to encourage civil society to participate in promoting mobility and road safety in the city. Since its inception, the Mobility Pact has facilitated initiatives and consensus on actions to improve mobility and urban road safety. Currently, nearly 100 associations, companies, bodies, and public entities related to mobility are involved in this pact.

The second Municipal Road Safety Plan, covering 2004–2007, integrated the Urban Mobility Plan for the first time. Its main objectives included: a European commitment to reducing fatalities by 50% between 2000 and 2010; decreasing the number of road traffic injuries in the city by 45% between 2003 and 2010; proposing a social agreement to achieve zero fatal crashes in Barcelona and joining 30 European cities in this commitment. Barcelona signed the European Road Safety Charter in 2004.

The main actions included addressing the behavior of users through education and enforcement; enhancing vehicle safety; improving infrastructure and traffic management; increasing safety in the professional transport of goods and passengers; improving assistance and first aid to injured individuals; and ensuring more accurate collection, analysis, and dissemination of data on crashes.

**Figure 2:**
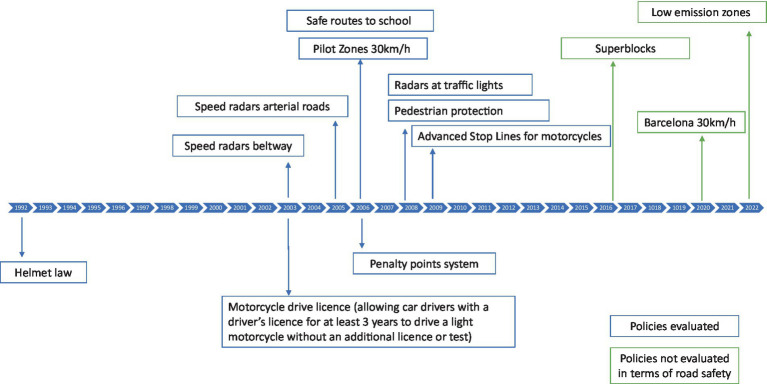
Timeline of Barcelona road safety interventions and evaluations (in blue).

Interventions were implemented in the city to achieve these goals, in line with the first Decade of Action on Road Safety 2011–2020, proclaimed by the UN General Assembly. These interventions included measures to reduce car use, such as raising parking costs and implementing traffic calming measures. Specific actions involved establishing 30 km/h zones, installing red-light cameras, creating bicycle lanes, and enhancing pedestrian protection with barriers, wider pavements, and safe routes to school. Figure 2 shows a timeline of major policies implemented in the city or brought about by national legislation (described later in this article) and evaluated.

Since then, additional road safety plans were developed, covering the periods 2013–2018 and 2018–2023, and linked to the city’s mobility plans. The most recent mobility plan for 2019–2024 is based on five main areas: safety, sustainability, health, equity, and smart mobility. This plan includes 62 lines of action and over 300 specific measures (10). Notably, it incorporates health as a new area of focus with 5 objectives.

## 3 Barcelona road traffic intervention effectiveness

Throughout the implementation of road safety policies in Barcelona, we assessed the effectiveness of several legislative and infrastructure interventions in terms of their impact on the number of collisions and injuries (Figure 2). The legislative policies evaluated include a helmet law (11), the penalty points system, and a motorcycle driver’s license (12). The infrastructure interventions assessed included speed radars (13, 14), 30 km/h speed limit zones, safe routes to school (15), advanced stop lines for motorcycles (16), and red-light cameras. We also estimated the cost–benefit ratio of speed radars (17). Table 2 summarizes the objectives, study designs, and main results of these evaluations conducted by the Agència de Salut Pública in Barcelona.

**Table 2:** Road safety interventions implemented and evaluated in Barcelona.

Evaluation study	Intervention	Objective	Design and data source	Main results
**Helmet law.** Impact of a helmet law on two-wheeled motor vehicle crash mortality in a southern European urban area (11)	A federal road safety law went into effect in the fall of 1992 extending to urban areas the unrestricted use of safety helmets by all two-wheeled motor vehicle users	To assess the effect of the law in reducing fatal motorcycle crash injuries; to estimate the number of lives saved; and to determine changes in the distribution of severity and anatomical location of injuries	Pre-test/post-test design to evaluate the effect of the helmet law by comparing data on traffic related deaths among two-wheeled motor vehicle occupants before and after implementation of the law Data sources: Barcelona Forensic Institute City police department Periods: Pre: 1990–1992 Post: 1993–1995	Decrease of 25% in the observed motorcycle crash mortality in the post-law period when compared with what would be expected if no such law had gone into effect Proportion of deaths with severe head injuries was reduced from 76 to 67% in the post-law period Prevented 35 lives of two-wheeled motor vehicle occupants
**Penalty points system** (Unpublished, conference presentation, SESPAS, 2007)	Penalty Points System (National strategy since July 2006)	To assess the effectiveness of penalty points system on the number of people injured in the Barcelona	Pre-post quasi-experimental evaluation design Data source: local police accident database Period: Pre: Jan 2002–Jun 2006 Post: Jul 2006–May-2007	Collisions: −6% Not Beltway: −8%. People injured: −6% (only significant for men, −9%) 15–35 years: −10% 36–50 years: −15% Car users: −8% Motorcyclists: −10% Men drivers: −9% 15–35 years: −10% 36–50 years: −12% Car users: −10% Motorcyclists: −16% Moped: −13% Non-significant for women drivers
**Motorcycle drive license** Road injuries and relaxed licensing requirements for driving light motorcycles in Spain: a time-series analysis (12)	A national law was passed in October 2004 allowing car drivers in possession of a driver’s license for at least 3 years to drive a light motorcycle (engine capacity 51–125 cc) without an additional license or test	To assess differences between the risk of injury for motorcycle riders before and after passing the law	A quasi-experimental study with comparison groups, using time-series analysis Intervention group: people injured while driving or riding a light motorcycle (engine capacity 51–125 cc) Comparison groups: People injured while driving or riding heavy motorcycles (engine capacity >125 cc), or mopeds (engine capacity £ 50 cc) or cars who were injured in a collision within the city limits Data source: local police accident database Period: Pre: Jan 2002–Sept 2004 Post: Oct 2004–Dec-2007	Number of people injured in a light motorcycle: +46% Number of people injured in a heavy motorcycle: +15% Attributable number had the law not been passed for the 3 years and 7 months of the study intervention: 2.091 light motorcycles users injured 691 heavy motorcycles users injured
**Speed Radars (Beltway)** Reducing Road Traffic Injuries: Effectiveness of Speed Cameras in an Urban Setting (13, 14)	Eight speed cameras went into operation on the beltway in March 2003 with the aim of reducing the number of road collisions and their consequences. The speed limit over most of the beltway is 80 km/h with some stretches limited to 60 km/h	To assess the effectiveness of speed cameras on Barcelona’s beltway in reducing the numbers of road collisions and injuries and the number of vehicles involved in collisions	Time-series quasi-experimental study with a comparison group. The “intervention group” was the beltway, and the comparison group were the arterial roads on which no fixed speed cameras had been installed Data source: local police accident database Period: Pre: Jan 2001–Mar 2003 Post: Apr 2003–Mar-2005	Decrease of 27% in number of collisions and number of people injured Decrease of 26% in number of vehicles involved in injury collisions. Greater effect during weekends (−34%) Prevented estimates for the 2 years of the study intervention: 364 collisions 507 people injured 789 vehicles involved in injury collisions
**Speed Radars (Arterial roads and beltway)** Effectiveness of speed enforcement through fixed speed cameras: a time-series study (14)	Speed cameras in the arterial roads (2005) and in the beltway (2003)	To assess the effectiveness of speed cameras in reducing the numbers of crashes and people injured of the arterial roads of Barcelona, and to assess their long-term effectiveness on the beltway	A time-series quasi-experimental design. on the arterial roads: the stretches of arterial roads encompassing 500 m before and after the location of the speed cameras were considered the enforced stretches of arterial roads. The remaining stretches of arterial roads being considered non-enforced arterial roads, (comparison group) Data source: local police accident database Periods: Arterial roads Pre: Jan 2002–Jul 2005 Post: Aug 2005–Dec-2007 Beltway: Pre: Jan 2001–Mar 2003 Post: Apr 2003–Dec-2007	Arterial roads: No observed changes Beltway: Decrease of 30% in number of collisions Decrease of 26% in number of people injured Prevented estimates for the 4 years and 5 months of the study intervention: 913 collisions 1,219 people injured
**Speed Radars (Cost–Benefit Effectiveness)** Speed cameras in an urban setting: a cost–benefit analysis (17)	Eight speed cameras went into operation on the beltway in March 2003 with the aim of reducing the number of road collisions and their consequences	To perform a cost–benefit analysis of the installation of speed cameras on the beltways of Barcelona	A cost–benefit analysis was performed from the society perspective over a 2-year period from April 2003 to March 2005	Base case results showed a net benefit of 6.8 million, ranging from €5.6 to €23.1 million, in the sensitivity analysis
**Advance Stop Lines for Motorcycles** Do advanced stop lines for motorcycles improve road safety? (16)	35 Advanced Stop Lines (ASLM) for motorcycles were implemented in 2009 (phase I) and 16 in 2010 (phase II)	To assess the effectiveness of ASLM for motorcycles in preventing road traffic collisions in phase I and phase II	A quasi-experimental study with comparison groups, using time-series analysis Included two study areas were: ASLM area and 30 m preceding (34 m); Crosswalk and intersection (CROSS) Data source: local police accident database. Periods: Phase I: Pre: 2002–2009 Post: 2010–2014 Phase II: Pre: 2002–2010 Post: 2011–2014	PHASE I 34M zone: Collision +34%, People Injured +26% Motorcycle involvement +44% Motorcycle driver involved +39% CROSS zone: No significant changes PHASE II No significant changes in either zone
**Safe Routes to School** Effectiveness of a road traffic injury prevention intervention in reducing pedestrian injuries, Barcelona, Spain, 2002–2019 (15)	Safe Routes to School (SRTS) intervention program carried out in Barcelona between 2006 and 2016	To assess the effectiveness of SRTS intervention in reducing the number of road traffic collisions and injuries in the school environment	Pre-post quasi-experimental evaluation design, with a matched comparison group, including 127 schools Data source: local police accident database Periods: Pre: Jan 2002–Mar 2006 Post: Apr 2007–Dec-2019	Number of collisions Involving 0–16 year-olds injured: −41.1% Involving 0–16 year-olds injured pedestrians: −43.3% People injured: Involving 0–16 year-olds injured: −36.6% Involving 0–16 year-olds injured pedestrians: −39% Annually prevented estimates for intervene schools: 16 collisions injuring 0–16-year-old 15 collisions injuring 0–16 year-old pedestrians 12 injured 0–16 year-old pedestrians Annually prevented estimates if SRT would have been extended to all schools: 97 collisions injuring 0–16 year-olds 90 collisions injuring 0–16 year-old pedestrians 75 injured 0–16 year-old pedestrians
**30 km/h Zones** (Unpublished, conference presentation, Encuentro de Ciudades 2011)	Speed zones at 30 km/h developed between Jan 2006–March 2007	To assess the effectiveness of implementation of speed zones at 30 km/h on the number of people injured by traffic collisions in the city of Barcelona	Pre-post quasi-experimental evaluation design with comparison group (close areas with similar characteristics) Data source: local police accident database Period: Pre: Jan 2002–Mar 2006 Post: Apr 2007–Dec-2010	People injured: Total: −12.2% Motorcyclists injured: −28.2% In street crossings: −18.5%
**Radars at traffic lights (PhotoRed)** (Unpublished)	Radars at traffic lights (10 radars at 30 settings) installed in 2008 (PhotoRed)	To assess the effectiveness of radars at traffic lights in the city of Barcelona	Pre-post quasi-experimental evaluation design with comparison group (all traffic lights without radars) in street crossings Data source: local police accident database Period: Pre: Jan 2002–Jul 2008 Post: Ago 2008–Oct-2010	Although the number of casualties at intersections with photored seems to decrease after the installation of the cameras, these differences are also observed at intersections of access roads that do not have photored, so it cannot be attributed to photored, but to other factors Time-series analysis, once adjusted, shows no significant change before and after the installation of photoreds in the number of people injured
**Overall interventions addressed to improve pedestrian road safety**	Handrails to protect pedestrians, traffic calming measures, identification of black points, interventions to reduce car use, better signalization of crossings	To estimate the number of injured pedestrians prevented from January 2008 to October 2011 considering the characteristics of the monthly series since January 2002	Pre-post time-series Data source: local police accident database Period: Pre: Jan 2002–Dec 2008 Post: Jan 2009–Dec 2011	Meant monthly reduction: −9-3% Number of prevented pedestrians injured (3 years and 8 months): −606 (or 158 annual)

### 3.1 Legislation evaluations

#### 3.1.1 Helmets

A national road safety law, enacted in 1992, mandated the use of safety helmets for all two-wheeled motor vehicle users in urban areas. Barcelona was the first Spanish city to strictly enforce the law, rigorously stopping two-wheeled motor users, imposing fines, and immobilizing vehicles until their drivers used a helmet. The evaluation showed a 25% decrease in motorcycle mortality 3 years after implementing the law compared with expected mortality without the law. The proportion of fatalities with severe head injuries decreased from 76 to 67%, and the law prevented an estimated 35 deaths among two-wheeled motor vehicle occupants (11).

#### 3.1.2 Penalty points system

Spain introduced the penalty points system as a national strategy in July 2006. After 11 months, injury-producing collisions were reduced by 6% in Barcelona city, with a reduction of 8% on the beltway. Injuries decreased by 6% overall and by 9% among men (more detail in Table 2). Age and gender analyzes revealed no significant effect among individuals older than 50 years, women, or collisions occurring outside the beltway. Despite the short follow-up, these results were consistent with an evaluation in Rome and the Lazio Region (18).

#### 3.1.3 Motorcycle license

A national law passed in October 2004 allowed car drivers in possession of a driver’s license for at least 3 years to ride light motorcycles (engine capacity 51–125 cc) without an additional license or test. An evaluation revealed that the law significantly increased traffic and injuries. The number of injuries in light motorcyclists increased by 46%, and those in heavy motorcyclists by 15%. In the 3-years and 7 months after the passing of the law, nearly 3,000 more riders were injured than before the implementation of the law (12).

### 3.2 Infrastructure evaluations

#### 3.2.1 Speed cameras

Speed cameras led to a significant 27% reduction in injury-producing collisions after 2 years of follow-up, which was maintained at 30% after 4.5 years, preventing 1,219 injuries (13, 14). A net benefit of €6.8 million was estimated (17). However, no effect was observed on arterial roads (14).

#### 3.2.2 30 km/h zones

The initial phases of implementing 30 km/h speed zones resulted in a 12.2% reduction in injuries, an 18.5% reduction at street crossings, and a 28.2% reduction in injuries to motorcyclists. This evaluation supported the expansion of 30 km/h speed zones to the entire city in 2020, except for the beltway and arterial roads, where speed limits are between 60 and 80 km/h.

#### 3.2.3 Safe routes to school

This intervention led to a significant 36.6% reduction in injuries among 0- to 16-year-olds and a 39.9% reduction among child and youth pedestrians. The intervention prevented an estimated 15 collisions injuring 0 to 16-year-olds, and 12 injuries to 0 to 16 year-old pedestrians annually (15).

However, not all interventions resulted in significant traffic injury reductions:

#### 3.2.4 Advanced stop lines for motorcycles (ASLM)

To facilitate mobility, a pioneering intervention was designed with no prior experience in other cities: implementing advanced stop lines for motorcycles (ASLM) without a specific lane to approach red lights, similar to bicycle lanes. The evaluation of the first phase showed a 34% increase in collisions, a 26% increase in injuries, a 44% increase in motorcycle involvement, and a 39% increase in motorcycle drivers involved in collisions in the space preceding the advanced stop line. Nonetheless, there were no significant changes in the space after the red lights at the crossing, which could have posed a risk for pedestrian collisions. The evaluation of the second phase of ASLM, implemented in a large arterial road, revealed no significant changes (16).

#### 3.2.5 Red-light radars

The evaluation of the effectiveness of installing 10 red-light radars in Barcelona in 2008, after 27 months of follow-up, showed no impact on the number of injured people, the number of drivers involved, or the number of injury-producing collisions. These results supported the decision not to extend red-light radars to the rest of the city.

## 4 How many injuries have been prevented due to road safety policies in Barcelona?

In this section, we discuss the overall impact of the interventions discussed above on road traffic injuries from January 2008, when most interventions had been implemented, until December 2023. The analysis was conducted by gender, injury severity, and mode of transport. We conducted a pre-post evaluation using a temporal series based on the Police Register of traffic crashes from the local police of Barcelona. The outcome variable was the number of injured individuals, analyzed using generalized linear models with a robust Poisson distribution. We adjusted for possible confounders, such as linear trend, seasonal patterns, and regression to the mean, using sine and cosine functions. The effects of the financial crisis since 2010 and the pandemic since March 2020 were also included. No adjustment was made for mobility exposure, as the interventions aimed to modify mobility itself. The aim was to assess magnitude trends rather than the risk of injury. For more detailed methods, see the Supplementary material.

### 4.1 Observed and expected road traffic injuries in Barcelona 2002–2023

**Figure 3:**
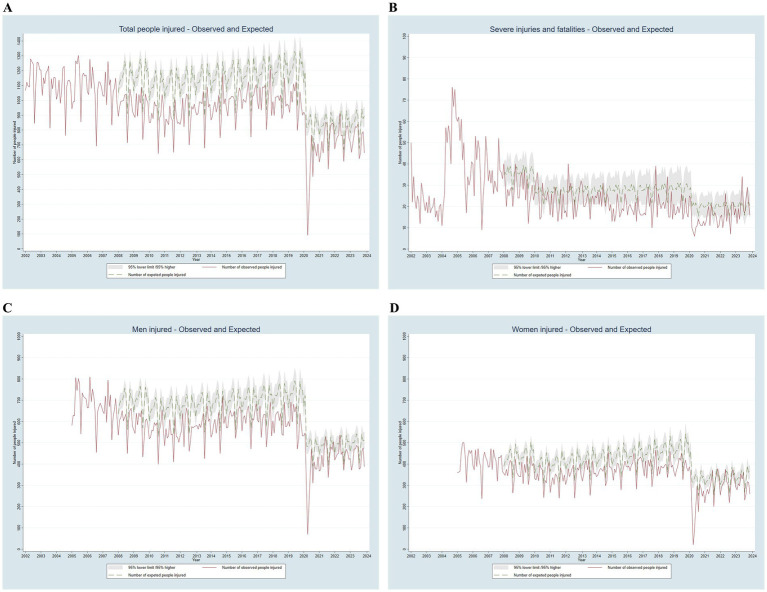
Observed and expected monthly number of people injured in road traffic collisions in Barcelona by severity and gender, adjusted for the financial crisis and COVID-19, 2002–2023.

**Figure 4:**
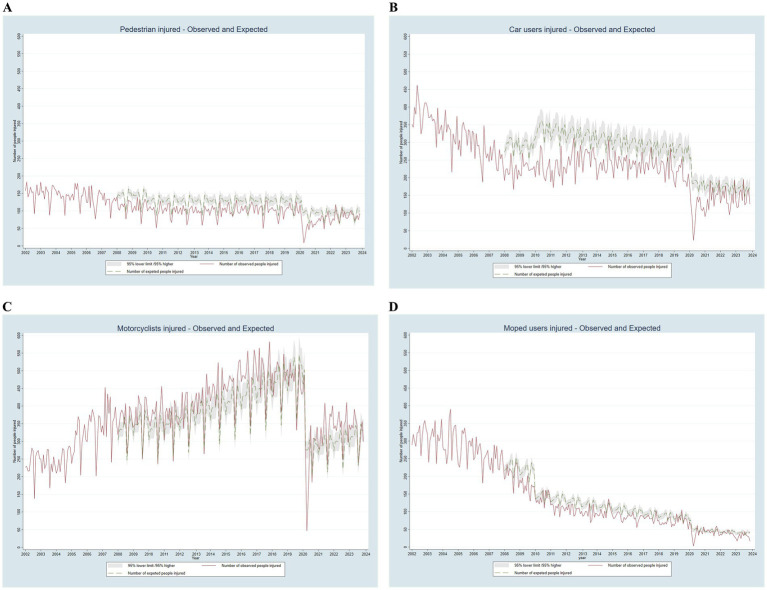
Observed and expected monthly number of people injured in road traffic collisions in Barcelona by mode of transport, adjusted for the financial crisis and COVID-19, 2002–2023.

Figures 3, 4 show the monthly distribution of observed (red line) and expected (dashed green line, with gray confidence intervals) numbers of people injured in road traffic collisions from 2002 to 2023, categorized by severity, gender, and mode of transport, assuming no interventions had been implemented. Except for motorcyclists, the number of expected injuries consistently exceeded the number of observed injuries. The total number of injuries decreased until 2011, then rose steadily until April 2020, when they fell sharply due to the COVID-19 lockdown. Severe injuries and fatalities decreased until 2010 and then stabilized, with an annual mean of 211 severe injuries and 23 fatalities.

The gender distribution followed a similar trend but with a lower magnitude for women (male/female ratio of 1.4 in more recent years). The trend for pedestrians remained highly stable. Car user injuries decreased until 2008, followed by a slight increase and then a stable pattern. Motorcyclists showed a continuous increase after 2005, following changes in light motorcycle driving license (12). In contrast, moped user injuries showed a continuous reduction.

According to the statistical model, since 2008 injury-producing road traffic collisions have been prevented in more than 34,800 individuals, including approximately 1,000 with severe injuries. By mode of transportation, injuries have been prevented in more than 4,700 pedestrians, 12,300 car users, and 3,200 moped users. However, there has been an excess of 5,200 injuries among motorcyclists (Table 3).

**Table 3:** Road traffic injuries observed, relative risk pre-post 2008, and prevented cases.

	Pre *n*	Post *n*	Mean (monthly) Pre	Mean (monthly) Post	RR*	RR* LL 95%CI	RR* HL 95%CI	% change	Prevented*	Prevented* LL 95% CI	Prevented* HL 95% CI
Total people injured	79,469	171,772	1103.7	894.6	0.833	0.792	0.873	−16.7	34,791	21,581	48,000
Severe injured and fatalities	2,505	4,021	34.8	20.9	0.782	0.658	0.930	−21.8	1,119	−67	2,306
Men +	24,368	104,673	676.9	545.2	0.849	0.807	0.894	−15.1	18,571	10,133	27,010
Women +	14,891	66,620	413.6	347.0	0.847	0.798	0.900	−15.3	12,002	5,681	18,323
**Mode of transport**											
Pedestrian	10,232	18,428	142.1	96.0	0.798	0.743	0.857	−20.2	4,674	2,494	6,854
Car users	23,028	40,052	319.8	208.6	0.764	0.709	0.824	−23.6	12,349	7,212	17,486
**Two wheel motor**											
Motocyclists	20,726	76,497	287.9	398.4	1.074	1.007	1.145	7.4	−5,249	−11,116	619
Moped users	20,632	17,772	286.6	96.7	0.848	0.799	0.901	−15.2	3,181	1,454	4,907

Pre: Pre period 2002–2007 (84 months); Post: Post period 2008–2023 (192 months).

RR: Relative Risk; LL 95%CI: Lower limit 96% Confidence Interval; HL 95%CI: Higher limit 96% Confidence Interval.

+ Data by gender is only available from 2005.

* Adjusted for finacial crisis and COVID-19.

## 5 Discussion

This study highlights the imperative of analyzing traffic injuries through an intersectoral approach, particularly from a public health perspective, given their significant scale. Evaluating the health impact of interventions is essential, as it enables the informed shaping of policies that extend beyond the health sector and profoundly affect the wider population. The article presents compelling evidence of the effectiveness of road safety interventions within urban settings, recognizing the distinct characteristics of space, roads, and transportation modes compared to interurban areas, which is a context with limited existing evidence.

Moreover, this study emphasizes the ongoing need to assess the health impact of policies under varying circumstances. Even interventions with established effectiveness may not consistently yield positive outcomes. As exemplified in this study, the influence of speed cameras on the beltway differed from their impact on arterial roads and intersections (e.g., red-light radars), illustrating the nuanced effects of interventions in diverse urban settings. This evaluation approach is essential for refining strategies and tailoring interventions to optimize their effectiveness in specific urban environments.

Nearly all the evaluations discussed in this paper were published in peer-reviewed journals, providing robust evidence of the effectiveness of these interventions. These findings align with other published experiences and suggest that similar strategies could be successfully implemented in other cities. However, the transferability of these interventions requires careful consideration of local contexts, as factors such as population characteristics, commuting patterns, and urban infrastructure can significantly influence outcomes (19). Therefore, while these interventions offer a valuable framework for road safety improvements, new implementations should always be evaluated to ensure they achieve the desired impact in different settings.

The estimated prevented injuries due to road safety policies in Barcelona over more than two decades is substantial, particularly severe injuries. Although being a rough estimation it aims to quantify effect of road safety policies to support further implementation. Some of the reduction since 2008 can also be attributed to the financial crisis, which appears to have affected car users more than motorcyclists. In addition to the financial crisis, policies to reduce car use, such as increased parking costs and traffic calming measures, may have led more people to switch from cars to motorcycles. Furthermore, some moped users might have transitioned to motorcycle use after he change in driver license law (12).

As conditions evolve over time, it is challenging to isolate and assess the effectiveness of each individual intervention over an extended period. Despite this limitation, the use of generalized linear models with a robust Poisson distribution provides a robust statistical framework for analyzing injury trends. This approach accounts for key confounders such as linear trends, seasonality, the financial crisis, and the COVID-19 pandemic, enhancing the reliability of the findings. Additionally, the analysis spans a broad period (2002–2023) and examines trends by severity, gender, and mode of transport, offering a comprehensive and nuanced understanding of road safety outcomes. However, as the study relies on observational data, it cannot establish direct causality, and unaccounted-for confounding factors may still influence the results. Several factors were important for success. Firstly, an essential factor was the recognition by public health professionals of road safety as a critical issue in health outcomes monitoring. Secondly, the early establishment of a comprehensive information system that integrated data from health and police sources proved instrumental in characterizing incidents, prioritizing road safety measures, and facilitating evaluation. Thirdly, effective collaboration and coordination among public health, police, and mobility professionals were achieved through a formalized agreement. Intersectoral collaboration for road safety in Barcelona involved regular coordination through joint committees, data sharing, and integrated action plans of mobility and road safety. The public health agency provided insights on injury trends, determinants, evaluation, monitoring and follow-up of action plans. In police role, there was enforced traffic laws and analysis of incident reports, and the mobility department focused on infrastructure improvements, and developing plans. Together, they allocate resources, engage the community for input (Mobility Pact) projects to test and refine strategies, such advanced zones for motorcycle. Continuous communication and monitoring ensure alignment, enabling to collectively reduce traffic-related injuries and fatalities while improving urban mobility.

Finally, civic engagement—evident in participation in the Mobility Pact, and more recently, the advocacy efforts of civil society groups—highlighted concerns about health impacts, particularly in road safety and air quality, thereby advancing road safety policies.

Several challenges mainly focused in developing a surveillance system and evaluating interventions merit attention. First, despite being well-documented for decades, significant gender-based inequalities in mobility patterns (20), modes of transportation, and consequently, the nature, frequency, and severity of injuries (21, 22), must be acknowledged. It is imperative to consistently incorporate a gender perspective into surveillance systems, data analysis, and the design and evaluation of interventions (23).

Second, another methodologically significant challenge in assessing road safety concerns the calculation of rates, which requires a denominator representing exposure to mobility (21, 22). While the resident population serves as a common denominator for numerous health issues, including country-wide analyzes of road safety, this approach is less practical at the city level due to the dynamic nature of population movement. To address this issue, precise denominators are essential, enabling risk analysis at smaller geographic levels and the inclusion of factors such as age groups, gender, mode of transport, and type of collision. This is important when implementing policies to reduce car usage, especially in a globalized society where, beyond daily commutes, individuals engage in extensive travel for tourism. Cities therefore experience substantial fluctuations in daily travel patterns, emphasizing the need for exposure denominators that reflect population movement.

Third, there is a pressing need to make progress in the interoperability of diverse data sources, likely linked through some form of common identifier. This would enable the severity and type of injuries to be studied, drawing from health sources, in conjunction with the circumstances of collisions, as reported by police sources. Harnessing the strengths of each source would provide a more comprehensive understanding of the impact of road incidents.

Fourth, it is essential to integrate the perspective of socioeconomic inequalities into road safety analysis (24). To achieve this, individual information on social class, educational level, place of residence, and the location of collisions is required. However, such information is often lacking in existing data sources due to data protection concerns. Moreover, collisions frequently occur outside individuals’ residential area due to their mobility (22). Addressing these challenges is vital for a comprehensive understanding of the impact of socioeconomic inequalities on road traffic injuries. It is highly recommended that this individual data becomes available for public health officers and researchers.

Fifth, while health records effectively document diagnoses and procedures—primarily due to their link with economic outcomes—details such as the external cause of the injury (e.g., frontal collision, pedestrian hit), mode of transport (e.g., bicycle, motorcycle), and position in the vehicle (e.g., driver, passenger) are often inadequately recorded. Although the ICD-10-CM provides a framework for detailed encoding, the absence of specific reporting or the use of non-specific codes poses a significant challenge. Comprehensive data on these factors is crucial for studying the nature and severity of injuries related to the vehicle involved and the circumstances of the collision, which would facilitate the design of preventive measures.

Sixth, intersectoral collaboration can be hampered by various political agendas. Road safety, framed within the “health in all policies” approach, requires a collaborative strategy that highlights the co-benefits for health, extending beyond the health sector to encompass multiple sectors. However, engaging these sectors has often proven challenging. In certain instances, policymakers have supported measures detrimental to health, often relying on overly narrow economic arguments that prioritize short-term gains for specific sectors over long-term societal benefits. Furthermore, some policymakers may misunderstand “Health in All Policies” as an expectation that health decision-makers resolve issues beyond their domain. Perceptions can be shifted by introducing the concept of “Health For All Policies” and emphasizing co-benefits, offering win-win solutions for all sectors (25). In Barcelona, for instance, health arguments have been effective in advocating for policies facing resistance, such as car restrictions within the city. Recognizing and integrating cross-sectoral action is imperative for advancing toward Sustainable Development Goals. Finally, evaluating all road safety policies and interventions, as with any health-related issue, is imperative. Determining their impact on health and health inequalities is crucial, as their effectiveness may vary among different territories and population groups. It is important to emphasize that the reported and evaluated interventions are the result of long-term planning and the gradual evolution of mobility in the city. These interventions were not all planned simultaneously but developed over time in response to emerging needs. Furthermore, the evaluations focused on interventions for which sufficient data were available, including adequate sample sizes, geographic distribution, follow-up data, and, when possible, comparable areas for controlling confounders. At the same time, they show the evolution of mobility policies in the city that gives priority to pedestrians, active mobility and reduces car use. The commitment to evaluation upholds the principles of transparency and accountability. Assessing the effectiveness of road safety interventions requires planning assessments alongside interventions, including comparison groups, and collecting exposure and outcome measures both before and after implementation. Additionally, it is vital to document interventions thoroughly, including details about the location and dates of implementation.

To strengthen the evidence base for policy-making, it is necessary conducting a more comprehensive cost–benefit analysis of road safety interventions in the future. Such an analysis would provide valuable insights into the economic and societal impacts of these measures, helping to identify the most cost-effective strategies for reducing road traffic injuries. By considering both direct costs, such as implementation and maintenance, and indirect benefits, including health outcomes and productivity gains, policymakers can make more informed decisions about allocating resources to maximize public health and safety outcomes.

In conclusion, the surveillance and evaluation of road safety interventions at the city level are pivotal for optimizing resources, tailoring strategies to local conditions, and continuously enhancing urban safety. This comprehensive 25-year assessment of road safety interventions in Barcelona reveals the complexity of implementing and appraising diverse strategies. The findings reveal a spectrum of effectiveness among interventions, with some demonstrating remarkable success and others falling short. This holistic evaluation approach, encompassing legislation, infrastructure, and economic considerations, provides indispensable insights for ongoing improvements and future road safety initiatives. These findings offer valuable insights to other cities but it needs to be approached with careful adaptation and additional evaluation.

A public health perspective serves as a guiding principle, ensuring a thorough understanding of the impacts of interventions on individuals, communities, and populations. Aligned with preventive and equity-focused principles, this approach contributes to the formulation of evidence-based strategies that promote and safeguard health and well-being. The estimation of prevented road traffic injuries in Barcelona serves as a testament to the effectiveness of the interventions implemented at the local level, providing a robust foundation for continual policy evaluation and refinement. These findings significantly contribute to the broader field of road safety and public health, offering invaluable insights for evidence-based strategies in injury prevention and advocacy for sustainable transportation modes at the city level.

## Data Availability

The raw data supporting the conclusions of this article will be made available by the authors, upon reasonable request. Requests to access these datasets should be directed to KP, cperez@aspb.cat.
