# Supplementary material for: 25 years of providing evidence on road safety interventions at the city level
Source: Front Public Health. 2025 Feb 7;12:1463878. doi: 10.3389/fpubh.2024.1463878 (PMC11844348; doi:10.3389/fpubh.2024.1463878)
Supplement: Supplementary file 1 [file Data_Sheet_1.docx]

**Supplemental Material**

**Annex 1. Estimates of prevented traffic injuries methods.**

This is a pre-post evaluation designed using temporal series. The source of information is the Register of traffic injuries of local police of Barcelona. Outcome variable was number of people injured due to traffic collision (including fatalities). Stratification variables were gender, age group, severity, and mode of transport. The study periods consider as pre-intervention: from January 2002 to December 2007, and post intervention: from January 2008 to December 2023.

As statistical analysis, first we did a descriptive analysis with comparison of monthly means with their interquartile and mean ranges, with their 95% confidence intervals in the pre- and post-intervention periods. Statistical significance has been verified with the Mann-Whitney nonparametric U test. This was followed by an analysis of time series of the result variable, using generalized linear models with robust Poisson distribution. Possible confounders such as regression to the mean, linear trend and seasonal patterns have been adjusted using sine and cosine functions, the effect of financial crisis since 2010, and the pandemic effect since March 2020. No adjustment for mobility exposure was done as it intended to assess magnitude trends, and not to assess the risk of being injured.

Once the trend and seasonal patterns were controlled, an intervention analysis was carried out by introducing a dummy variable to compare the pre- and post-intervention periods (before and after January 2008). Thus, the model can be summarized as follows: ln[E(Yt)]=B+B1×t+B2×sin(2 t/T)+πB3×cos(2 t/T)+B4×πXt, where T is the number of periods described by each sinusoidal function (e.g., T=12 months), t is the time period (t=1 for January, t=2 for February, etc.), and Xt = 1 to identify the post-intervention period and X_t_ = 0 for the pre-intervention period. The models have been adjusted for overscattering by scaling standard errors using the square root of Pearson's Ji-square. Relative risks (RR) have been calculated from the adjusted models. The analysis was carried out with STATA v14.

The expected number of injured has been calculated considering the monthly series of the number of people injured from January 2002 to December 2007 and its confidence interval at 95%. The number of injured prevented is determined from the difference between the number of injured expected and the actual number observed.
